# The Challenge of Preventing Cardiovascular Disease in Tunisia

**Published:** 2005-12-15

**Authors:** Hassen Ghannem

**Affiliations:** Department of Epidemiology, University Hospital Farhat Hached

## Abstract

Chronic disease, and particularly cardiovascular disease (CVD), is the major cause of death in most developed countries, despite the downward trend observed during the last three decades. Although CVD is emerging in developing countries, little is known there about comprehensive preventive measures for controlling its expansion.

The health care system in Tunisia faces the challenge of increasing rates of CVD risk factors. Epidemiologic studies show high levels of CVD risk factors among Tunisian adults and children. Evidence shows that several risk factors and conditions are commonly associated with major chronic diseases. Integrated actions against selected risk factors (i.e., smoking, physical inactivity, and unhealthy diet), implemented within the social context, can lead to the reduction of major chronic diseases. These interventions should take place early in childhood.

In Tunisia, a much-needed community-based intervention program to control CVD is being planned. This program will promote healthy living, smoke-free air, healthy nutrition, regular physical activity, and supportive living and working environments. Its ultimate goal is to reduce the burden of CVD and its related behaviors. A description of this program and how it will be implemented and assessed in the region of Sousse, Tunisia, is presented.

## Introduction

Chronic disease, particularly cardiovascular disease (CVD), is the major cause of death in most developed countries (1,2), despite the downward trend observed during the last three decades (3-5). The risk factors for chronic disease are also well known in most industrialized countries (6-11), and knowledge of risk factors has led to implementation of effective preventive programs (12,13). Although CVD is emerging in developing countries, little is known about the level of CVD risk factors in these countries (14,15). The problems of chronic disease are more serious for developing countries because many of them have not yet conquered communicable diseases, and their health systems are ill prepared to provide the costly care required for chronic diseases. Despite the new interest in and emphasis on public health and disease prevention in developing countries, it appears that the challenge of controlling CVD remains.

Tunisia is now facing the phenomenon of epidemiologic transition (16): total mortality is decreasing, life expectancy is increasing, and lifestyles associated with chronic disease, particularly diabetes and CVD, are being adopted (17,18). With this transition, the health care system in Tunisia is challenged with the expansion of chronic disease. Environmental and behavioral changes — such as new dietary habits, the lack of physical activity, and the stresses of urbanization and work conditions — can lead to the rise of CVD and its risk factors.

The major CVD risk factors — high blood cholesterol, high blood pressure, cigarette smoking, physical inactivity, and unhealthy diet — satisfy the public health criteria of causality (19). In fact, strong epidemiological evidence suggests that these risk factors explain at least 75% of new cases of coronary heart disease (CHD) each year. Available evidence supports the feasibility and effectiveness of population-wide prevention programs directed toward increasing the proportion of people at low risk for CVD. The public health effort should be directed to this population-based approach (20). Evidence shows that several risk factors and conditions are commonly associated with major chronic diseases. This means that integrated actions against selected risk factors (i.e., smoking, physical inactivity, and unhealthy diet) implemented within the social context can lead to the reduction of major chronic disease; the Countrywide Integrated Noncommunicable Diseases Intervention (CINDI) illustrates this idea at work (21).

In Tunisia, a much-needed community-based intervention program to control CVD is being planned. This program will promote healthy living, smoke-free air, healthy nutrition, regular physical activity, and supportive living and working environments. Its ultimate goal is to reduce the burden of CVD.

## The Burden of CVD in Tunisia

In the context of epidemiologic transition and disease prevention, it is important to be able to assess the scope of existing CVD, but there is a lack of systematic monitoring of CVD morbidity and mortality in Tunisia and in most of the developing world. In Tunisia, the only available population-based epidemiologic data is a profile of CVD risk factors (22-24). In 1996, we conducted an epidemiological survey of a representative household sample (n = 957) of the adult urban population in Sousse. The objectives of the study were 1) to estimate the prevalence of the primary CVD risk factors in an urban context and 2) to collect data that would serve as a baseline for assessing future trends in risk factors. We observed a high prevalence (18.8%) of hypertension (blood pressure greater than 160/95 mm Hg); 28.8% had blood pressure greater than 140/90 mm Hg. Of the survey participants, 10.2% had a history of diabetes and 27.7% were obese (body mass index [BMI] ≥30), with a significantly higher rate among women (34.4%). The rate of android obesity (i.e., distribution of fat concentrated in the center of the body) was 36.0%, and the rate of smoking was 21.5%, with a significantly higher rate among men (61.4%).

Based on this profile of CVD risk factors, Tunisia can be compared with Western societies and should consider a national strategy of primary prevention and heart-health promotion in addition to the efforts recently made in secondary prevention of some chronic diseases such as hypertension and diabetes.

Urbanization is expected to raise the level of CVD risk factors in Tunisia as a result of the adoption of new dietary habits, the lack of physical activity, and the stresses of working conditions in urban areas. Changes in dietary habits have not yet reached a critical point, however; a study published in 1999 showed that the food regimen in Tunisia was still based mainly on carbohydrates, and the percentage of lipid calories did not reach 20% of total intake (25).

The assessment of CVD risk factors during childhood is also important because the underlying process of developing CVD starts early in life. The assessment of CVD risk factors before adulthood could inform us about the etiology of CVD. The results would serve as basic information in a public health context, notably in the promotion of healthy lifestyles. In 1999, we conducted a study of students aged 13 to 19 years; the study was partially funded by the Tunisian Ministry of Higher Education, Scientific Research and Technology (26) and was based on a representative sample of 1569 youths in Sousse to assess the following CVD risk factors: hypertension, diabetes, hypercholesterolemia, dyslipoproteinemia, obesity, lack of physical activity, and smoking. The study established local reference values for the percentile distribution of CVD risk factors. The study showed that BMI, diastolic blood pressure, total cholesterol, low-density cholesterol, and high-density cholesterol were significantly higher for girls than for boys. However, boys had significantly higher levels of systolic blood pressure. In addition, 7.9% of the study population was obese; a greater percentage of girls (9.7%) was obese than boys (6.0%). Overweight was also significantly higher for girls (16.1%) than for boys (11.1%). Smoking was reported by 7.6% of the population; the percentage of boys who smoked (14.7%) was significantly higher than for girls (1.1%).

## Effectiveness of a Community-based Intervention Program

Available evidence supports the feasibility and effectiveness of population-wide prevention directed toward increasing the proportion of people at low risk of developing CVD (19). CVD risk factors can be linked directly to social, economic, and environmental determinants of health. Factors that have a major impact on the development of chronic diseases include education, availability and affordability of healthy foods, access to health services, and infrastructures that support a healthy lifestyle (27). Advances in etiological research of CVD have resulted in numerous intervention projects and programs throughout the developed world. The scope of these activities is wide, from preventive action on a single risk factor (e.g., tobacco use, hypertension) or on a single disease (e.g., CHD) to a more comprehensive approach involving several risk factors common to several chronic diseases (21). These programs have clearly demonstrated the feasibility of comprehensive community-based cardiovascular programs and the need to extend intervention activities to other chronic diseases. The North Karelia project in Finland is a good example of how a demonstration project can be expanded to a national level (28).

For developing countries, evidence supports the benefits of lowering risk distributions. For example, the Asia Pacific Cohort Studies Collaboration indicates that a 2% reduction of mean blood pressure has the potential to prevent 1.2 million stroke deaths annually (approximately 15% of all stroke deaths) and 0.6 million coronary deaths (6% of all CHD deaths) by the year 2020 (29). Much more attention should also be directed to modifying the environmental determinants of physical inactivity and the resulting obesity (30).

One could expect a long-term positive impact of a community-based intervention program to control CVD in developing countries, based on the accumulated experiences of developed countries. The CVD epidemic is not a matter of fate in developing countries; it can be controlled or at least postponed in a country with a transitional economy, such as Tunisia. Because communicable diseases have not yet been conquered, the health care system in Tunisia is ill prepared to provide the costly care required for managing chronic diseases, but some efforts have been made recently. A national program was launched in 1993 by the Ministry of Health; this program was designed to improve the quality of care for patients with diabetes and hypertension in primary care settings. Its main objective was to standardize the management of these two CVD risk factors with a focus on general physician training and health education. This program is insufficient, however, and needs to be completed with a primary prevention approach, which is the main objective of our current initiative.

## Steps to Launch a Community-based Intervention Program

In Tunisia, we are in the initial stages of documenting the CVD burden and identifying its risk factors. This phase should be followed by a population-based preventive intervention to control the expansion of CVD. The potential for prevention generated by a community-based intervention program is important, and so is the idea of building collaboration among different categories of professionals toward a common goal of CVD prevention. In Tunisia, the launch of such an intervention would represent a cornerstone for a chronic disease control program. To launch the intervention, we should adopt a stepwise approach, described below.

### Step 1: community mobilization and assessment

In this phase, we will focus on community mobilization and establish a task force of politicians, health professionals, and nongovernmental organizations committed to decreasing CVD in Sousse. This stage will also include a community assessment with the following parameters: levels of CVD risk factors; knowledge, attitudes, and behaviors related to CVD risk factors; patterns of use of primary health services; and barriers to and facilitators of healthy behavior.

### Step 2: intervention planning and implementation 

First, the task force will use the community assessment data for setting priorities. Second, educational and skill-building activities will be coordinated through existing social and organizational structures such as primary health care centers, work settings, secondary schools, health clubs, and community recreational facilities.

### Step 3: sustainability and evaluation

This phase will focus on the sustainability and evaluation of the intervention based on its feasibility and impact on CVD in Sousse. Evaluation is a continuous, systematic process of determining what has been achieved in a program and comparing these achievements with the objectives set out in the plan. Evaluation should not take place only at the end of the program; it must be considered in the planning phase of the program, as soon as program activities begin, and should continue until after the activities have ended. Components of evaluation should include the following:

- **Implementation.** This component systematically examines what is delivered and how it is delivered. It does not focus on program results but provides documentation of program delivery.
- **Impact.** This component assesses the intervention's ability to produce positive changes in levels of risk factors and other determinants of CVD (e.g., physical activity, BMI, healthy diet, knowledge, attitudes, behaviors).
- **Outcome.** This component assesses the effectiveness of the intervention in producing long-term changes in CVD among the target population of Sousse.

## Integrated Program to Control CVD in Sousse

Sousse is the fourth largest city in Tunisia, with a total population of 547,000, according to the most recent census in 2004. There are 92 primary health care centers and two university hospitals. The total number of primary health care physicians is 168; 92 physicians are in the private sector and 76 are in the public sector. There are 57 secondary schools with a total of 1762 classes and 56,752 students. Sousse has 413 worksites that employ more than 50 employees each.

### Description of the program

The integrated program will target youths as well as adults through lifestyle-education activities within the general framework of community mobilization. Improving the preventive practices of health professionals at different levels of care will also be central to the program. The program will address the entire community (from symptom-free individuals to high-risk individuals) and propose interventions centered on promoting healthy habits (e.g., smoking abstinence, balanced nutrition, sustained physical activity) and preventing main risk factors (e.g., arterial hypertension, diabetes, hypercholesterolemia) toward the ultimate goal of reducing or delaying CVD.

The program focuses not only on interventions targeting internal factors that are controlled by each individual but also on the external environmental factors not individually controlled. The effectiveness of interventions on behavior modification (adoption of healthy habits) represents our primary challenge.

### Program objectives

The program objectives include a 10% relative improvement in healthy habits in 5 years in the target population of Sousse, including the following:

- 10% reduction in the proportion of sedentary adults
- 10% reduction in the prevalence of adult smokers 
- 10% reduction in the prevalence of young smokers 
- 10% reduction in the prevalence of obese adults 
- 10% increase in the proportion of the adult population consuming 5 daily servings of fruits and vegetables

The program will be implemented gradually with the following objectives:

- To implement the intervention annually among 30% of the physicians in the private sector and 30% of the physicians in the public sector
- To implement the intervention annually among 30% of worksites with more than 50 employees
- To implement the intervention annually among 30% of the secondary schools 

Other objectives may be added during implementation as we adapt to the process; for example, we may add other target groups such as pharmacists and nurses.

### Program strategies

The program will emphasize an integrated approach that combines two strategies: educational actions and environmental actions.

**Educational actions**

The educational actions will aim to encourage physicians and other primary health care professionals to introduce a brief counseling session to patients on the healthy habits related to CVD risk factors. For physicians, workshops of continuing medical education will focus on managing CVD risk factors in primary care.

**Environmental actions**

The environmental actions will aim to modify the environments of work settings, schools, and the general community. Three types of environments are targeted: physical, economic, and social. At worksites, we will actively engage the support of members of management and occupational health and medicine groups. We plan to approach a new group of schools each year during the program so that by the end, we reach all schools in the region. In the community, we will focus on leisure services and sports facilities to help them develop and implement public policy to modify environments to promote the adoption of healthy habits. The promotion of healthy lifestyles in these environments is intended to create smoke-free environments, encourage smoking cessation, promote the consumption of fruits and vegetables, and encourage regular physical activity. These dimensions are summarized in the Figure.

**Figure:**
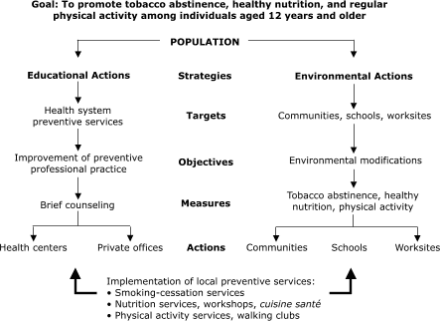
Integrated program of chronic disease control, Sousse, Tunisia.

## Conclusion

Tunisia is a country with a transitional economy that faces the challenge of an increase in morbidity associated with chronic disease. With this epidemiologic transition, the health care system must be prepared to address the growing expansion of chronic disease, particularly CVD. Many epidemiologic studies have demonstrated high levels of CVD risk factors among Tunisian adults and children. The time for prevention is now. There is evidence that several risk factors and conditions are commonly associated with major chronic diseases. This means that integrated actions against selected risk factors (smoking, physical inactivity, and unhealthy diet) implemented within the social context can lead to the reduction of major chronic disease. Prevention of chronic disease should focus on decreasing risk factors early in childhood. The community-based program in Tunisia will target access to positive healthy living, smoke-free air, healthy nutrition, regular physical activity, and supportive living and working environments. Its ultimate goal is to reduce the burden of CVD. The implementation of this program is a public health priority and will serve as a useful example for other developing countries.
